# The meninges as a neuroimmune interface: structure, barriers and roles in CNS disease

**DOI:** 10.1186/s12987-026-00795-5

**Published:** 2026-03-17

**Authors:** Sarah Joost, Hannes Kaddatz, Elise Vankriekelsvenne, Lars-Ove Brandenburg

**Affiliations:** 1 Institute of Anatomy, Rostock University Medical Center, Rostock, Germany; 2 Department of Neurology, Rostock University Medical Center, Rostock, Germany; 3 Centre for Transdisciplinary Neurosciences Rostock (CTNR), Rostock University Medical Center, Rostock, Germany

**Keywords:** Meninges, Dura mater, Arachnoid mater, Pia mater, Barrier, Meningeal immunity

## Abstract

The meninges form a highly specialized barrier and immune interface that protects the central nervous system (CNS), regulates cerebrospinal fluid (CSF) dynamics, and coordinates communication between the CNS and the periphery. Each layer—dura mater, arachnoid mater and pia mater—possesses distinct structural, vascular and immunological features that collectively shape CNS homeostasis. A broad range of anatomical and molecular studies has revealed that meningeal compartments are far more heterogeneous and functionally complex than traditionally recognized, particularly with respect to their barrier architecture and immune interactions. In this review, we summarize current knowledge of meningeal structure and function, with a focus on barrier properties and immune-cell trafficking. We further discuss how meningeal dysfunction contributes to pathology in bacterial meningitis, multiple sclerosis and Alzheimer’s disease. Emerging evidence highlights the meninges as an active neuroimmune organ rather than a passive covering, critically influencing inflammation, solute clearance and disease progression. Understanding these mechanisms may open new therapeutic avenues targeting meningeal pathways across neurological disorders.

## Introduction

**Fig. 1:**
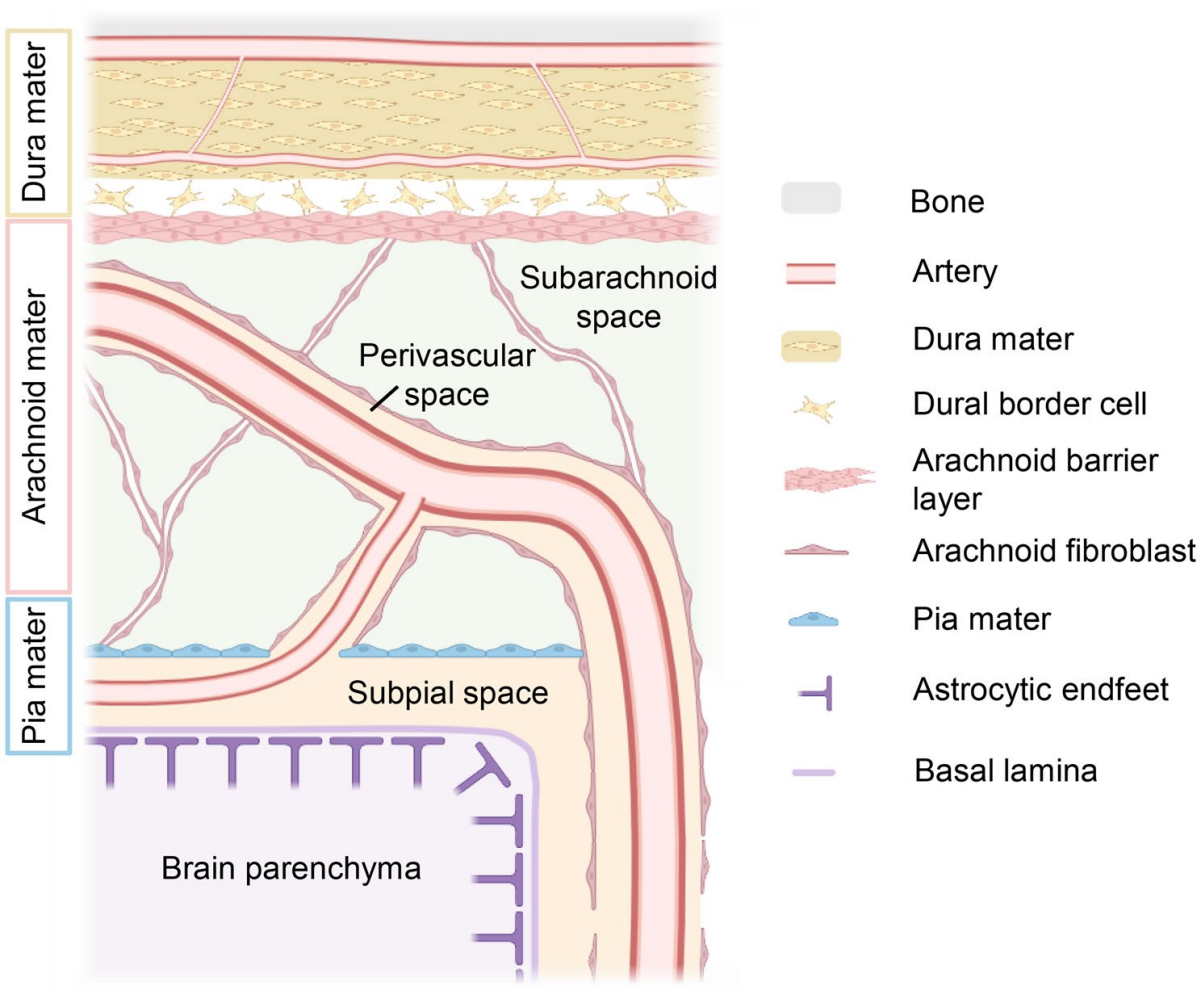
Meningeal architecture. The dura mater is a multilayered fibroblast and collagen-rich structure with embedded blood vessels, lymphatic vessels (not shown), and a layer of dural border cells opposing the arachnoid mater. The arachnoid mater consists of an upper arachnoid barrier cell layer and arachnoid trabeculae that span the subarachnoid space. Leptomeningeal blood vessels traverse the subarachnoid space, are covered with leptomeningeal cells and eventually penetrate the brain parenchyma. The single cell layer of the pia mater is continuous to arachnoid cell populations and covers the subpial space. Below the subpial space, the glia limitans of the brain parenchyma built of a basal lamina and astrocytic endfeet is located. Created with http://www.Biorender.com

The Central Nervous System (CNS) is enveloped by three distinct layers of connective tissue, collectively known as the meninges. These layers provide structural protection and are classified as the dura mater (tough outer layer), the arachnoid mater (delicate, spiderweb-like middle layer), and the pia mater (thin, innermost layer). The arachnoid and pia mater together form the leptomeninges, which enclose the cerebrospinal fluid (CSF) within the subarachnoid space (Fig. 1) [1].

Alongside the skull and vertebral column, the meninges and subarachnoid space play a crucial role in shielding the CNS from mechanical forces, infections, and other forms of injury. Beyond their protective function, the meninges are also essential for brain development and are implicated in various pathological processes [1, 2]. Bacterial and viral infections (meningitis), primary tumors (meningiomas), and metastatic malignancies affecting the meninges are life-threatening conditions [3–6]. Additionally, hemorrhages resulting from vascular injury above or within the meninges can lead to hematoma formation, posing a severe risk. These and other aspects of meningeal pathophysiology have been extensively reviewed in the literature [7–9].

This review aims to summarize and compare current knowledge on the structure and function of the dura, arachnoid, and pia mater, with a particular focus on their role as a barrier. Additionally, we explore meningeal immunity and the migration of immune cells across the meninges. Building on this foundation, we analyze the involvement of the meninges and their associated structures in CNS diseases, including bacterial meningitis and neurodegenerative disorders such as Multiple sclerosis (MS) and Alzheimer’s disease (AD). A more comprehensive understanding of meningeal functions may provide valuable insights into CNS pathology and contribute to the development of novel therapeutic approaches.

## Dura mater: structural framework and immunological interface

As the outermost meningeal layer, the dura mater is composed of fibroblasts within a robust collagen-rich extracellular matrix. Although often portrayed as a simple structural sheath, anatomical and molecular studies reveal a highly specialized tissue harboring a complex extracellular matrix architecture, a dense and heterogeneous vascular system, functional lymphatic vessels, and extensive sensory and autonomic innervation.

Classically, the dura mater is described to consist of two layers: the periosteal (or endosteal) layer and the meningeal layer. The periosteal layer corresponds to the periosteum of the skull bone. The meningeal layer is closely adjacent to the periosteal layer within the brain cavity but separates from the vertebral periosteum in the spinal canal, thereby creating the fat-filled epidural space. Invaginations of the meningeal layer form the dural folds – the falx cerebri, tentorium cerebelli, falx cerebelli and diaphragma sellae – which provide mechanical stabilization of the brain. Venous blood is collected in dural sinuses situated between the periosteal and meningeal layers and ultimately drained toward the jugular vein. At the ultrastructural level, the dura mater can be further subdivided into several (up to five) distinct layers that differ in collagen fiber organization, vascular density and cellular composition [10, 11].

Directly beneath the dura mater, a layer of dural border cells is located. Compared to dural fibroblasts, dural border cells exhibit a more branched morphology and are not interconnected by tight junctions [12]. The intercellular space forms relatively large open clefts that are devoid of collagen fibrils but instead contain a finely granular extracellular matrix [13]. This region has been referred to by different terms in the literature, most commonly as the dural border cell layer or the subdura. In contrast to the mechanically robust dura mater and arachnoid mater, the subdura represents a structurally weak interface that is prone to separation under non-physiological mechanical stress [14].

The dura mater is vascularized by three meningeal arteries per side (anterior, middle and posterior meningeal arteries) which run along its outer, periosteal surface together with corresponding veins. From these vessels arises an extensive vascular network that spreads across the dural surface and gives rise both to arteries penetrating the skull and to branches descending into deeper layers of the dura. Within these deeper layers – only a few micrometres above the dural border – an additional dense capillary bed is formed, extending throughout the dura mater, including its folds [15, 16]. In contrast to leptomeningeal vessels, dural blood vessels are fenestrated and lack tight junctions, features consistent with roles in immune surveillance and CSF drainage [17–19].

**Fig. 2:**
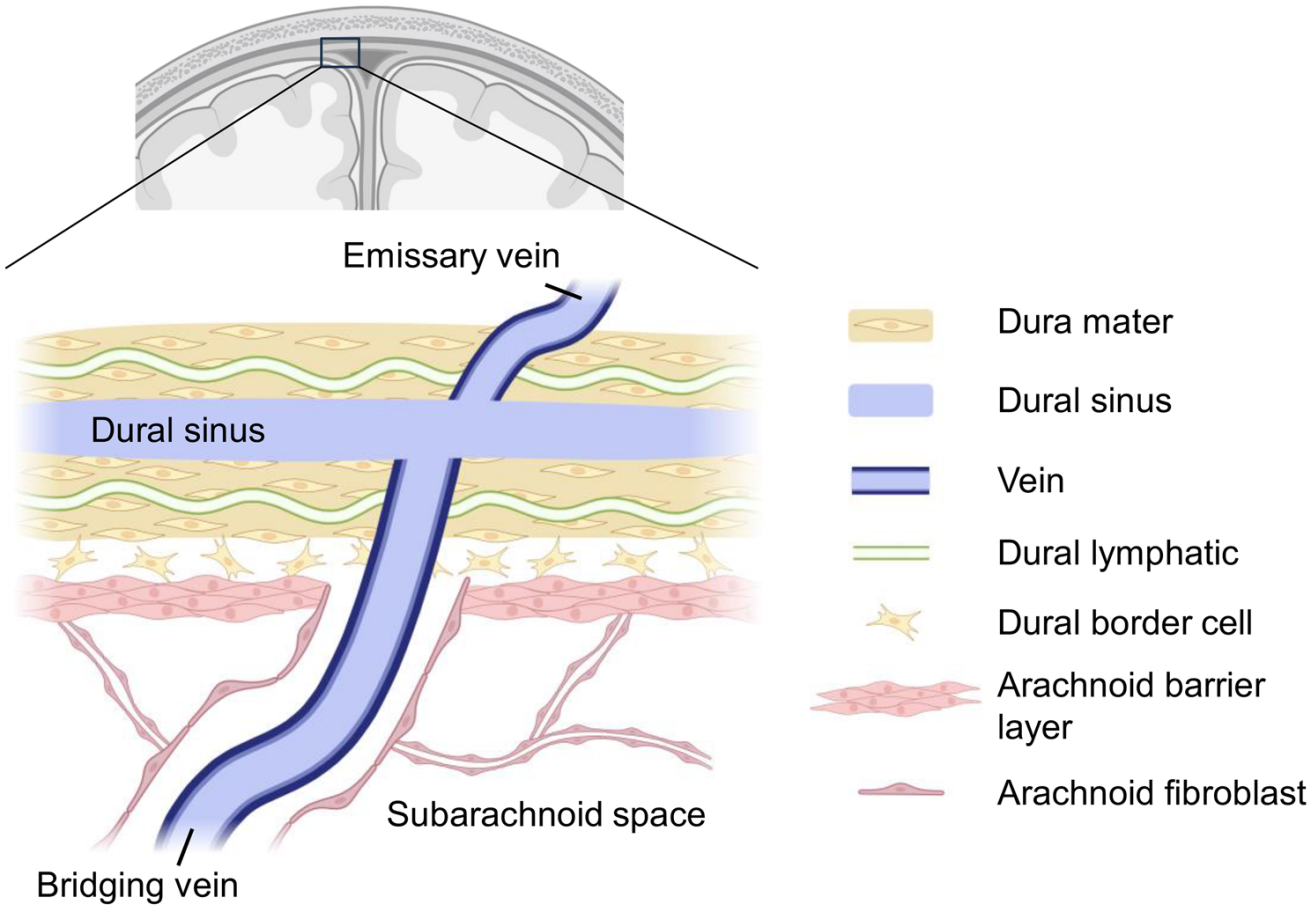
Connection of dura mater, dural sinus and leptomeninges. The dura mater contains lymphatic vessels and blood vessels (not shown) and encloses the dural sinuses. The arachnoid barrier layer of the leptomeninges is directly opposed to the dural border cell layer of the dura mater. Bridging veins, covered by leptomeningeal fibroblasts in the subarachnoid space, traverse the dura mater to connect with the dural sinuses. Additionally, emissary veins link the dural sinuses to veins within the skull’s bone marrow cavity (not shown). Created with http://www.Biorender.com

Notably, the dural sinuses are linked to the superficial venous system by emissary veins [20] which pass the skull through osseous channels to reach the bone marrow cavity [21–23]. These emissary veins are connected to diploic veins within the spongious substance of the calvaria, which in turn are connected to extracranial veins of the scalp [24]. Intracranially, the dural sinuses are further connected to subarachnoid veins *via* bridging veins [20]. Together, these venous pathways create a continuous vascular network linking subarachnoid veins, bone marrow venous channels and superficial extracranial veins (Fig. 2). Importantly, several studies have demonstrated that bone marrow-derived cells such as granulocytes [22], monocytes [25] or B cell precursor cells [26] can migrate *via* these small vascular connections into the intracranial space and penetrate the meninges. This recruitment is proposed to occur *via* migration along the extracellular matrix of emissary and bridging veins, mediated by interactions between α6-integrin and laminin [27].

In addition to its vascular network, the dura mater also contains lymphatic vessels. Although the existence of a dural lymphatic system was long debated, it is now firmly established through several landmark studies [28, 29]. Dural lymphatic vessels express characteristic markers of lymphatic endothelial cells, including vascular endothelial growth factor receptor (VEGFR) 3, Prospero homeobox protein 1 (Prox1), podoplanin (gp38), lymphatic vessel endothelial hyaluronan receptor (Lyve1), CD31, and CCL21 [29–31]. These vessels possess a discontinuous basement membrane and are primarily located along the superior sagittal and transverse sinuses, as well as in lateral and basal regions of the skull [32, 33]. Meningeal lymphatics vessels (mLVs) are evolutionarily conserved and have been identified in humans, nonhuman primates, rats, mice, and even fish [7]. Functionally, the dural lymphatic system contributes to drainage of CSF, metabolic by-products and CNS-derived antigens toward the deep cervical lymph nodes, either directly or indirectly [34], as extensively reviewed elsewhere [29, 35]. In addition, arachnoid granulations at the dura-brain interface have been proposed to act as adhesive pockets where CNS antigens are trapped and immune cells are sequestered, functioning in a manner similar to lymph nodes [36].

The dura mater also exhibits extensive sensory innervation, which is provided by the meningeal branches (*rami meningei*) of various cranial and cervical nerves. The meningeal branches of the glossopharyngeal and vagus nerves innervate the dura of the posterior cranial fossa, excluding the clivus region. The meningeal branch of the mandibular nerve (V3) supplies the parietal dura up to the falx cerebri, and the maxillary nerve (V2) innervates the dura of the middle cranial fossa. The ophthalmic nerve (V1) contributes to innervation of the anterior cranial fossa, while cervical spinal nerves (C1-C3) primarily innervate the clivus region [37, 38].

Autonomic innervation of the dura is dominated by sympathetic fibers originating from the superior cervical ganglion. These fibers reach the cranial dura *via* periarterial plexuses accompanying the meningeal arteries, most notably the middle meningeal artery and the internal carotid artery. In contrast, parasympathetic innervation of the dura mater remains poorly characterized. Although cranial parasympathetic ganglia – such as the ciliary, pterygopalatine, and otic ganglia – may modulate vascular tone and nociceptive processing, definitive anatomical evidence for direct parasympathetic projections to the dura is still lacking [39, 40].

Notable interspecies differences in meningeal anatomy have been documented and may help explain some controversial descriptions of meningeal structure. For example, comparative histological analyses of the dura mater show that species vary in dural thickness, number of fibrovascular layers, and fibroblast orientation; whereas humans have distinct periosteal and meningeal dural layers, other mammals such as rats or sheep may exhibit a single fibrovascular layer with different cellular organization [41]. Additionally, while meningeal lymphatic vessels have been observed in both humans and non-human primates, the architecture and imaging feasibility can differ across species, suggesting that insights from rodent models do not necessarily translate directly to primates [42]. These species-specific structural and cellular variations in the meninges—including differences in barrier properties, vascular and immune composition—highlight the need for caution when extrapolating anatomical or immunological features from animal models to the human CNS.

## Arachnoid mater: barrier layer and subarachnoid organization

**Fig. 3:**
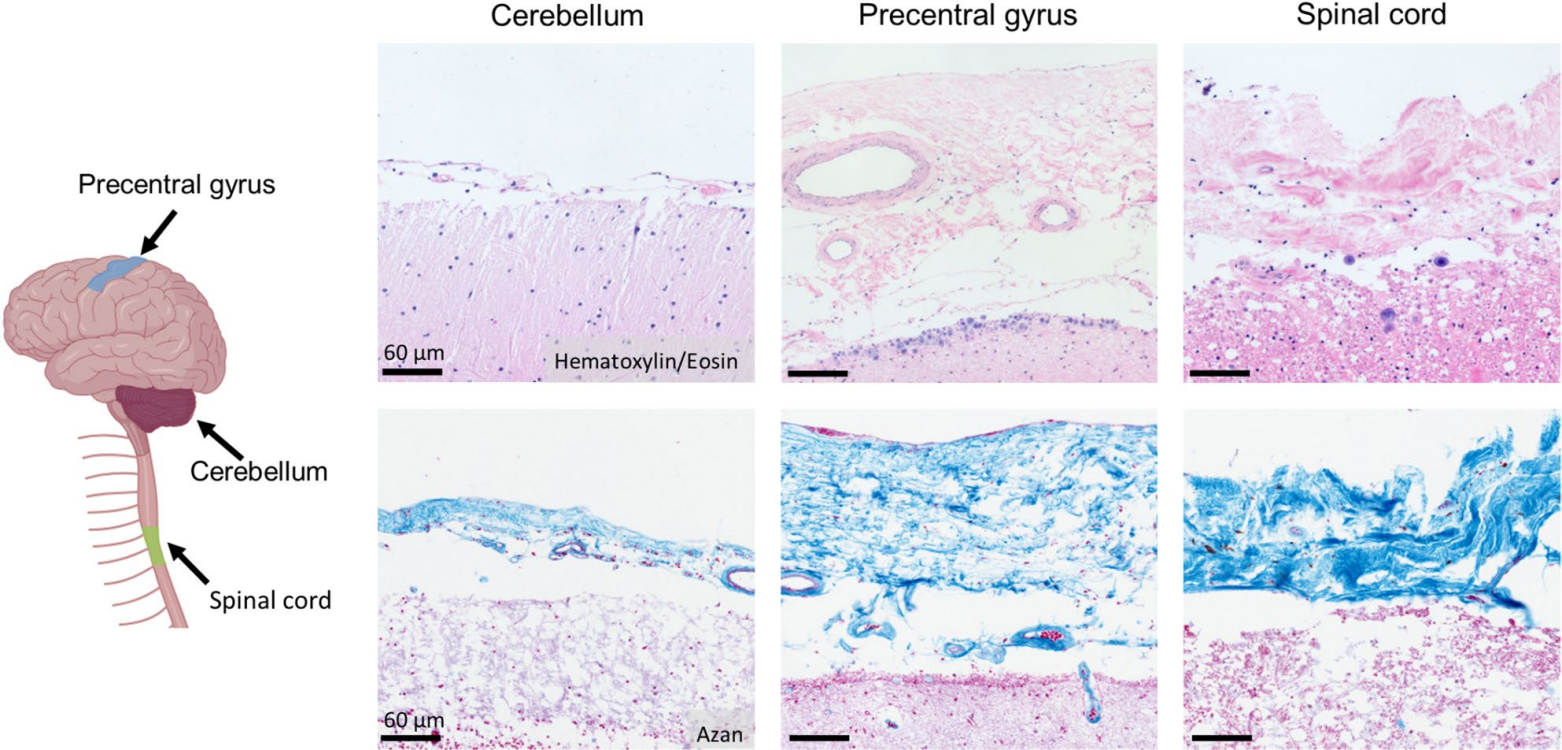
Histological characteristics of the human leptomeninges. The thickness of the human leptomeninges varies across different regions of the central nervous system. Shown here are histological sections of post mortem leptomeninges in the cerebellum, precentral gyrus, and spinal cord. The upper row displays hematoxylin/eosin staining, in which both brain parenchyma and leptomeninges appear pink. The lower row shows azan staining, where the leptomeninges stain blue, contrasting with the pink-stained brain parenchyma. Created with http://www.Biorender.com.

The intermediate meningeal layer, the arachnoid mater, encloses the subarachnoid space containing CSF between the arachnoid and the innermost meningeal layer, the pia mater (Fig. 3). In contrast to the dura mater, the arachnoid mater is a thin, translucent, and avascular membrane. Structurally, it can be subdivided into an outer mesothelial layer, an intermediate layer, and a deeper layer of loosely packed cells with numerous collagen fibers [43]. Arachnoid trabeculae, composed of fine connective tissue, extend between the arachnoid and the pia mater, connecting both layers and giving the subarachnoid space its characteristic spiderweb-like appearance.

The outer surface of the arachnoid mater adheres to the dural border cell layer of the dura mater [44]. At this interface, the arachnoid mater forms a specialized neurothelium composed of several layers of flattened, tightly interconnected cells [45]. These neurothelial cells represent modified fibroblasts. The structure is variably referred to as the arachnoid barrier cell layer, arachnoid neurothelium, or lamina neurothelialis. Functionally, the arachnoid barrier cell layer acts as a diffusion barrier between the vascularized dura mater and the CSF-filled subarachnoid space, contributing to the separation of these compartments [46]. In vivo, the arachnoid is firmly attached to the dural border cell layer *via* this neurothelial interface [47]. However, postmortem degradation of the arachnoid–dural interface results in layer separation and detachment from the dura mater, producing an artifactual cleft that has historically been interpreted as a subdural space [13, 14]. A true subdural space does not exist under physiological conditions; it forms only pathologically. For instance, injury to bridging veins traversing these layers can result in venous bleeding into the mechanically weak dural border cell layer causing widening of the subdura and therefore artificially creates a subdural space [48].

The arachnoid mater spans across the cerebral gyri, allowing the subarachnoid space to expand into various cisterns. These cisterns are topographically defined but not anatomically distinct. They are interconnected by a trabeculated, porous structure with variably sized openings. In neurosurgical practice, these cisterns serve as important landmarks for orientation and surgical access to specific deep brain regions [49].

Arachnoid trabeculae are specialized connective tissue structures that mechanically and biologically integrate the arachnoid and pia mater, thereby contributing to subarachnoid space stability, cerebrospinal fluid dynamics, and the regulation of cellular migration within the meningeal compartment [50]. The arachnoid trabeculae are composed of fibroblasts that produce type I collagen fibrils and additional extracellular matrix components that form the structural core of the trabeculae [50]. In addition, substantial amounts of collagen type II have been described within the leptomeninges, which may contribute to the loosely organized, mesh-like architecture of the trabecular network [51]. The collagen-rich network of arachnoid trabeculae may provide a structural scaffold that regulates immune-cell migration and retention in the subarachnoid space and may support compartmentalized meningeal inflammation and tertiary lymphoid structure formation in neuroinflammatory diseases such as multiple sclerosis [51]. The plasticity of extracellular matrix components in neurological disease has recently gained increasing attention; however, the mechanisms underlying remodeling and structural reorganization within the meninges remain incompletely understood [52]. Accordingly, the role of arachnoid trabecular architecture in shaping immune cell behaviour, as well as its remodeling under pathological conditions, remains largely unexplored.

Arachnoid villi, or arachnoid granulations, are specialized structures of the arachnoid mater that protrude into the dura and dural venous sinuses, facilitating CSF drainage into the venous system [53]. Despite extensive study, their precise functional relevance remains unclear. Historically, it was believed that CSF outflow primarily occurred *via* arachnoid granulations into the dural venous sinuses. Today, no universally accepted model explains the exact mechanism of CSF transport through these structures [54].

Recent work challenges the traditional view of the meninges as a three-layered structure. Studies by Møllgård [55] and Plá [56] propose a fourth meningeal layer that may act as a permeability barrier, functionally dividing the subarachnoid space into inner and outer compartments, distinct from the arachnoid barrier. However, the existence and role of this putative fourth layer remain the subject of intense ongoing debate [57, 58].

The subarachnoid space contains a vascular network of both arteries and veins. Leptomeningeal arteries, arising from the internal carotid and vertebral arteries forming the circle of Willis, spread across the cortical surface and within sulci, branching repeatedly to form an extensive network down to diameters of approximately 30–40 μm [59]. These arteries penetrate the brain parenchyma, where they form the capillary beds. In contrast, cerebral veins emerge from the parenchyma into the subarachnoid space, where they converge into larger veins that drain into the dural sinuses via bridging veins. Consequently, the subarachnoid space contains arteries and veins of various calibers, but no capillaries. Both arterial and venous vessels in the subarachnoid space are encased in a leptomeningeal cell sheath, which is continuous with the pia mater [60]. These ensheathing cells are thought to derive from both the pia mater and the arachnoid mater and are therefore commonly referred to as leptomeningeal cells. However, their precise characterization remains ambiguous. While they are frequently described as fibroblasts or fibroblast-like cells, their circumferential ensheathing organization and the presence of desmosomal junctions [12] and keratins [51] also suggest epithelial-like properties. Between the vessel adventitia and these leptomeningeal cells, a perivascular space exists, which can expand under pathological conditions, e.g., due to immune cell accumulation [61, 62].

The leptomeninges also form attachment sites for the choroid plexus, the CSF-producing tissue located within all ventricles. Notably, the stroma of the choroid plexus is continuous with the subarachnoid space [63] and anchores the choroid plexus to the arachnoid compartment at multiple sites [64], establishing a close spatial relationship between the ventricular lumen and the subarachnoid space. It remains a topic of discussion whether an alternative pathway for CSF flow from the ventricles to the subarachnoid space exists *via* these contact regions, particularly under pathological conditions such as obstructive hydrocephalus [65–68].

## Pia mater: structure, perivascular architecture and possible immune entry routes

The third and innermost meningeal layer is the pia mater, a delicate, highly vascularized membrane that directly envelops the brain and spinal cord, forming a firm attachment to the underlying neural tissue. The cranial pia mater closely follows the brain’s contours, covering the gyri and extending into fissures and sulci [69]. Histologically, the pia mater consists of a single- to double-layered sheet of leptomeningeal cells. These cells possess long, thin projections and are primarily connected by desmosomes and gap junctions. The outermost epipial layer contains collagen fibers, while the inner intima pia is composed of elastic and reticular fibres [69, 70]. The pia mater rests upon the basement membrane of the glia limitans, from which it is separated by a subpial space containing collagen bundles, fibroblast-like cells, and blood vessels. Structurally, the cells of the epipial layer are connected to the arachnoid *via* arachnoid trabeculae, while the intima pia adheres tightly to the outermost layer of neural tissue, the glial membrane.

A key feature of the pia mater is its formation of vascular sheaths around blood vessels entering and exiting the CNS perpendicularly to the meningeal surface. This arrangement creates a fluid-filled space between the vessel walls and the pia mater, known as the perivascular space or Virchow-Robin space [71]. However, the detailed architecture and molecular composition of this boundary layer separating blood vessels from brain tissue remain incompletely understood [72].

The leptomeningeal sheath and its associated perivascular space extend along penetrating arteries down to the capillary level, where endothelial cells fuse with the glial basement membrane, effectively obliterating the space. A similar, but shorter, perivascular compartment accompanies veins as they exit the parenchyma [69]. Around cortical arteries, the perivascular space appears to be mostly compact and minimally open under physiological conditions [73]. In contrast, perivascular spaces in the basal ganglia and white matter are more open and structurally distinct. Here, leptomeningeal cells form two concentric layers around vessels, with the perivascular space located between them [60, 74].

A longstanding point of debate concerns whether perivascular spaces are continuous with the subarachnoid space. Ultrastructural evidence indicates that the pia mater forms a continuous sheath on the CNS surface that reflects along penetrating vessels, completely enclosing them in a leptomeningeal cell layer [60, 61, 70, 73]. Under this model, the perivascular space connects to the subpial –but not the subarachnoid – space; a concept supported by some MRI studies in humans [75, 76]. However, other studies report tracer exchange between subarachnoid and perivascular compartments or fail to reproduce these morphological barriers, suggesting at least partial continuity between the subarachnoid space and perivascular spaces [65, 77, 78]. Of note, ultrastructural analyses constitute the primary source of data addressing these morphological questions, as they provide the necessary spatial resolution. However, such approaches are inherently susceptible to artifact generation and are often limited in statistical power. Tracer studies face similar limitations, as injections into confined spaces may disrupt fragile membranes and thereby introduce structural distortions. Consequently, the question of continuity between meningeal compartments remains of high importance, particularly for understanding immune cell entry, solute exchange, and pathogen spread between meningeal and perivascular compartments.

Among the different perivascular compartments, the Virchow-Robin space surrounding penetrating vessels is especially prominent. It represents a critical anatomical interface linking the vascular system, CSF dynamics, and CNS immune surveillance. Although the Virchow-Robin space is separated from the subarachnoid space, various substances – and under certain conditions, pathogens – can move between these compartments, influencing the progression of infections and inflammatory responses. For leukocytes, perivascular spaces represent not merely transit zones to the CNS parenchyma, but dynamic immunological niches that can initiate or amplify neuroinflammation. Other functions of the perivascular space include its role as a conduit for cerebrospinal fluid leading to the draining lymphatic vessels [79, 80].

The glia limitans forms the direct interface between the pia mater and the CNS parenchyma, serving as a crucial structural and functional boundary. The entire surface of the brain and spinal cord, including the perivascular spaces, is enveloped by this specialized glial layer. The glia limitans creates a barrier at all CNS parenchymal surfaces, including the outermost layer (*glia limitans superficialis*) and the perivascular surfaces (*glia limitans perivascularis*). Structurally, it consists of a parenchymal basement membrane formed by astrocytes and their endfeet. In the healthy CNS, water transport across this barrier is regulated by the polarized expression of aquaporin-4 (AQP4) in the astrocyte endfeet, facilitating fluid exchange between the perivascular spaces and the neuropil. Beyond its role in fluid regulation, the glia limitans also functions as an immunological barrier, restricting the uncontrolled infiltration of immune cells from the subarachnoid and perivascular spaces into the CNS parenchyma. Astrocyte endfeet within the glia limitans are interconnected by gap junctions, allowing communication between astrocytes and contributing to the coordinated regulation of homeostasis [81–84].

## Meningeal immunity and immune cell migration

The meninges constitute an immunologically active compartment that harbors a diverse set of resident immune cells. Single-cell studies have identified a broad panel of resident immune populations in the meninges. In mice, these include dendritic cells, monocytes, macrophages, natural killer (NK) cells, and both B and T cells [85]. Human leptomeninges show a comparable diversity, with three subsets of border-associated macrophages, tissue-resident CD4^+^ and CD8^+^ T cells, and additional monocyte and B-cell populations [86]. Both dural and leptomeningeal immune populations undergo continuous turnover from bone marrow-derived precursors, indicating active exchange with the peripheral immune system [25, 26]. This implies a role in CNS immune surveillance. Beyond classical immune functions, several studies surprisingly link leptomeningeal immune cells to behavioural phenotypes in mice; depletion of these populations alters learning, social interaction and stress responses [7, 87–90]. In the context of neuroinflammation, the leptomeninges are considered a potential site for immune cell migration [91], accumulation [92], activation [93] and expansion [94]. Consistent with this, intravital microscopy in murine models has visualised T cells exiting arachnoid vessels into the subarachnoid space [95]. In the murine experimental autoimmune encephalomyelitis (EAE) model, meningeal inflammation was detected before symptom onset [96] and depletion of meningeal immune cells has been shown to reduce neuroinflammatory responses in models of stroke and subarachnoid hemorrhage [97, 98]. For comprehensive overviews of meningeal immunity, we refer to several reviews [7, 99, 100]. In the following, we focus on the anatomical routes of immune-cell migration involving the meninges and illustrate their relevance in three representative disease contexts: bacterial meningitis, MS, and AD.

## Immune cell migration via the meninges

The meninges serve as a key interface for immune surveillance of the CNS. Under neuroinflammatory conditions, they mediate two principal forms of immune trafficking: (i) export of CNS-derived antigens or antigen-presenting cells to peripheral lymph nodes, and (ii) the entry of peripheral immune cells into the CNS.

Dural lymphatic vessels drain towards the deep cervical lymph nodes, and both CNS antigens and antigen-presenting cells carrying CNS-derived material have been observed to reach these nodes, particularly under neuroinflammatory conditions [101–105]. The migration of CNS antigens to peripheral lymph nodes can activate or prime adaptive immune responses [106], potentially marking one of the earliest steps in autoimmune responses targeting the CNS, such as in multiple sclerosis. The precise mechanisms by which antigen-presenting cells and antigens migrate from the brain parenchyma to the leptomeninges and cross the arachnoid barrier to access dural lymphatics remain unclear. However, Smyth et al. [107] recently proposed that CSF and immune cells may cross the arachnoid barrier alongside bridging veins, at sites they termed ‘arachnoid cuff exit points’. These structures represent regions where the arachnoid mater forms a cuff-like extension around bridging veins, creating local discontinuities or thinning of the barrier that may permit controlled exchange between the subarachnoid and dural compartments. Their discovery suggests that communication across the arachnoid barrier is more spatially organized than previously assumed and, if confirmed, could provide a mechanistic link between leptomeningeal immune activity and dural lymphatic drainage pathways.

Peripheral immune cells can access the CNS through several anatomical routes, including the blood-brain-barrier, the blood-CSF barrier, and the blood-meningeal-barrier (reviewed in [18, 108]). The relative contribution of each pathway varies across disease states and remains incompletely understood. Migration of immune cells *via* the meninges into the CNS includes multiple aspects. For one, immune cells of the calvaria bone marrow can migrate into the dura mater as described above. These dural immune cells are proposed to access the leptomeninges, potentially *via* perivascular pathways along bridging veins as described by Smyth et al. [107]. However, the specific mechanisms enabling these cells to traverse the arachnoid barrier remain unknown.

**Fig. 4:**
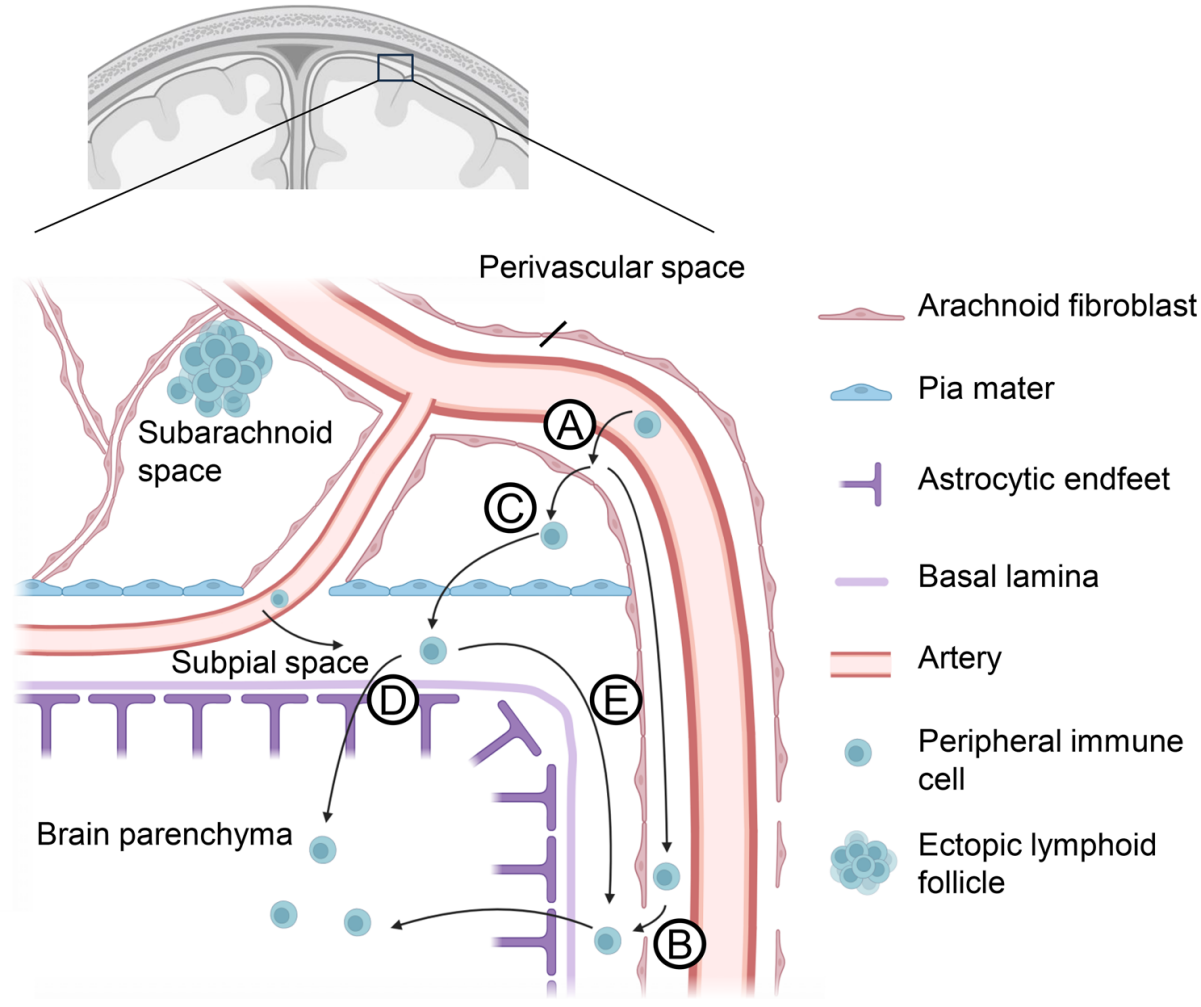
Migration routes from leptomeningeal vessels into the brain parenchyma. Leptomeningeal vessels in the subarachnoid space are ensheathed by leptomeningeal fibroblasts, creating a perivascular space between the vessel wall and the ensheathing fibroblast layer. As blood vessels penetrate the pia mater, either extending into the subpial space or entering the brain parenchyma, the arachnoid fibroblast layer transitions seamlessly into the pia mater. Consequently, the subarachnoid and subpial spaces remain distinct compartments. Potential migration routes for peripheral immune cells from meningeal blood vessels (**A**) into the brain parenchyma—beyond direct crossing of the blood-brain barrier in parenchymal vessels—include the perivascular space (**B**), the subpial space (**D**, **E**), and the subarachnoid space (**C**), as indicated in the figure. Created with http://www.Biorender.com

A better-characterized route for peripheral immune cell entry is extravasation from leptomeningeal blood vessels (Fig. 4). In neuroinflammatory mouse models, T cells adhere to leptomeningeal blood vessels, breach the endothelial blood–brain barrier, and enter the perivascular space (Fig. 4A) [95, 109]. From there, they may either migrate along perivascular pathways into deeper brain regions (Fig. 4B) or cross the leptomeningeal barrier to enter the subarachnoid space (Fig. 4C). Once within the subarachnoid compartment, immune cells can, in principle, reach the CNS parenchyma through two major routes: (i) trans-pial migration, involving passage across the pia mater, the subpial space, and the superficial glia limitans to access superficial cortical layers (Fig. 4D), and (ii) perivascular migration, whereby cells travel along the perivascular spaces of penetrating vessels to deeper cortical or subcortical regions before crossing the perivascular glia limitans into the parenchyma (Fig. 4E). The precise anatomical origin of perivascular migration remains debated. It is uncertain whether penetrating-vessel perivascular spaces communicate primarily with the subarachnoid compartment, the subpial space, or with perivascular spaces of leptomeningeal vessels [60, 61, 65, 70, 73–78, 110]. The figures in this review assume communicating subpial and perivascular spaces, separated from the subarachnoid space. Moreover, the restrictiveness of the membranes separating these spaces, and whether immune cells can cross them under physiological or pathological conditions, remains unresolved.

## Immune and barrier dysfunction of the meninges in bacterial meningitis

The meninges play a pivotal role in the pathogenesis of bacterial meningitis, serving both as the initial site of infection and the primary interface between invading pathogens and the CNS. Depending on patient age, *Streptococcus pneumoniae*, *Neisseria meningitidis* and *Haemophilus influenzae* type B represent the predominant causative agents. Clinically, patients typically present with fever, headache and neck stiffness, with progressive impairment of consciousness as inflammation escalates [111]. Untreated bacterial meningitis is frequently fatal and survivors often experience long-term neurological sequelae [112, 113].

Bacteria may enter the CNS directly after traumatic brain injury, or, more commonly, after colonizing mucosal surfaces such as the nasopharynx and subsequently invading the bloodstream [114]. Following bacteraemia, several pathogens, including meningococci and pneumococci, can cross the blood-brain barrier to reach the subarachnoid space. The precise strategies used to traverse meningeal capillaries remain incompletely understood and are the focus of ongoing research [115]. It also remains unclear which exact anatomical route bacteria take to reach the meninges after leaving the bloodstream. The perivascular or Virchow-Robin spaces appear to play an important role in this process [116]. In any case, bacterial meningitis leads to an enlargement of the perivascular spaces [117], accompanied by a massive accumulation of bacteria and, subsequently, peripheral immune cells [116]. During pneumococcal meningitis, the glymphatic system is disrupted due to the detachment of astrocyte endfeet from the vascular endothelium of the blood-brain barrier, impairing the function of the aquaporin 4 water channel and likely contributing to perivascular space enlargement [118]. The resulting reduction in glymphatic clearance leads to the accumulation of neurotoxic bacterial products in the cerebrospinal fluid spaces, ultimately causing widespread neuroinflammation and neuronal damage with consequent impairment of neurological function [119, 120].

After gaining access to the subarachnoid space, bacteria rapidly expand and spread along the leptomeningeal surfaces. Leptomeningeal cells express various surface receptors that recognize bacterial adhesins, facilitating attachment and colonization [121]. Weller and colleagues provide a comprehensive summary of known receptor-ligand interactions between human leptomeningeal or meningioma cells and bacterial adhesins, particularly those of meningococci [110]. Proposed mechanisms for traversing the leptomeningeal layer include intracellular penetration and junctional degradation, such as desmosome disruption [110, 122]. In advanced or fatal stages of meningitis, widespread death of leptomeningeal cells exposes collagenous pia mater and the glia limitans superficialis, potentially triggering microglial activation [110].

Once within the CSF, bacteria encounter a niche that, despite relatively low concentrations of glucose and proteins compared to plasma, supports rapid proliferation [123]. Several pathogens upregulate virulence factors in response to CSF exposure, promoting persistence and inflammation [124, 125]. Clinically, reduced CSF glucose levels reflect active bacterial metabolism within the subarachnoid space [126]. CSF flow likely facilitates bacterial dissemination throughout the leptomeningeal compartment. In contrast, the dura mater, which lacks this dynamic CSF environment, may constitute a less favorable environment for bacterial proliferation. In addition, structural features of the arachnoid, including arachnoid trabeculae, may create microenvironments that support bacterial adhesion and focal colonization, although this remains to be experimentally confirmed [100, 121, 127].

Bacterial meningitis also profoundly affects meningeal and cortical vasculature. In animal models, infection induces transcriptional alterations in leptomeningeal endothelial cells, leading to redistribution of tight-junction components such as claudin-5 and platelet and endothelial cell adhesion molecule (PECAM-1; CD-31) and compromising blood-brain barrier-integrity [128]. Meningococci, in particular, bind to endothelial surfaces via type IV pili, enhancing their resistance to shear stress and promoting transendothelial traversal [129, 130].

The cumulative effect of bacterial invasion and vascular disruption triggers a massive inflammatory response, characterized by recruitment of peripheral immune cells, particularly neutrophils, and activation of resident innate immune cells, including microglia and astrocytes. The leptomeninges contain numerous macrophages, fibroblasts, mast cells and plasma cells that contribute to cytokine and chemokine release, further amplifying the inflammatory cascade [131]. Neutrophils migrate from the bloodstream into the leptomeninges at sites of infection, where they mediate bacterial killing but may also contribute to tissue damage [132].

Future studies should aim to delineate the mechanisms of immune-cell recruitment and mobilization within the meninges, as well as the pathways by which infection spreads along perivascular spaces into brain tissue. A deeper understanding of how blood-brain barrier-associated cells and meningeal cell populations interact with bacteria and shape inflammatory responses may reveal new therapeutic opportunities.

## Meningeal inflammation in multiple sclerosis

MS is a chronic autoimmune disorder of the CNS characterized by demyelination, inflammation, and axonal degeneration. The disease involves the migration of peripheral immune cells into the CNS and their subsequent targeting of the myelin sheath, leading to impaired neural conduction. Clinically, MS presents with heterogeneous symptoms, including motor and sensory deficits, visual disturbances, and cognitive impairment. Despite intensive research, key aspects of MS pathogenesis – such as the initial trigger of the autoimmune response and the precise migratory routes of peripheral immune cells – remain incompletely understood [133].

Leptomeningeal involvement in MS was first highlighted through observations of increased immune-cell infiltration in post-mortem leptomeningeal tissue from MS patients [134–138]. Approximately 40% of examined *post mortem* brains of patients with secondary progressive MS exhibit leptomeningeal lymphoid follicles – organized aggregates of T cells, B cells, plasma cells, and follicular dendritic cells resembling germinal centers of secondary lymphoid organs [139–144]. Within these lymphoid follicles, clonally expanded B cells have been identified, indicating local B-cell activation and diversification and therefore their possible involvement in disease processes [145].

Meningeal inflammation appears to play a central role in cortical pathology in MS. It is closely associated with the development of subpial cortical lesions and the severity of cortical demyelination [146, 147]. Notably, cortical damage exhibits a gradient that decreases from the meningeal surface towards the inner parenchyma [148, 149]. The extent of meningeal inflammation correlates with clinical disability, cortical neurodegeneration, and overall disease progression [137, 141]. Experimental findings from a rat model of meningeal inflammation further support these observations, demonstrating that meningeal inflammation leads to cortical microglial changes closely mirroring those observed in post-mortem MS tissue [150].

Clinically, MS patients experience an increased prevalence of headaches, including migraines and tension-type headaches [151–154]. However, the causal relationship between MS and headache remains unclear, partly due to challenges in distinguishing between primary headaches and secondary headaches caused by MS [155]. Additionally, some pharmacological MS therapies may induce headaches as a side effect [156]. It has been hypothesized that meningeal inflammatory activity preceding a relapse could provoke headaches, potentially serving as an early sign of relapse, though this hypothesis remains unproven [157].

Together, these findings highlight the meninges as an active immunological niche in MS, where sustained inflammatory activity might contribute to cortical neuroinflammation and degeneration. The presence of ectopic lymphoid structures and associated immune-cell activation underscores the importance of meningeal-parenchymal interactions in disease progression. Understanding how meningeal inflammation arises and propagates may therefore be key to identifying new therapeutic targets for MS.

## Meningeal clearance pathways and vascular dysfunction in Alzheimer’s disease

AD is the most common cause of dementia and a major neurodegenerative disorder, characterized by the accumulation of misfolded proteins in the brain, including extracellular amyloid-beta (Aβ), particularly Aβ_1−42_, which forms amyloid plaques [158]. Although meningeal changes in AD remain comparatively underexplored, three components have recently gained increasing attention: (i) alterations of glymphatic and meningeal lymphatic clearance pathways, (ii) vascular and perivascular changes, including cerebral amyloid angiopathy, and (iii) cellular alterations within meningeal cell populations with emphasis on immune cells. Their precise contributions to AD pathogenesis, however, remain poorly understood.

The role of the meninges in AD is most often discussed in the context of mLVs and their contribution to Aβ clearance. Dural mLVs drain interstitial and CSF-derived solutes, including Aβ [159]. Aging-related degenerative changes in these vessels restrict efficient metabolite clearance, negatively affecting CNS homeostasis and potentially contributing to AD pathogenesis [160]. Enhancing meningeal lymphatic function is therefore considered a promising therapeutic strategy for improving Aβ clearance [161–163]. While impaired lymphatic drainage represents one mechanism promoting extracellular Aβ accumulation, perivascular pathways also appear to play a significant role.

In addition to reduced lymphatic clearance, AD is associated with deposition of Aβ within the perivascular spaces of leptomeningeal and cortical arteries – a condition known as cerebral amyloid angiopathy [164, 165]. Extensive Aβ accumulation weakens vessel walls, obstructs perivascular spaces, and impairs interstitial fluid flow, often accompanied by dilation of these spaces [164]. These vascular and perivascular alterations may disrupt local microenvironments at the meningeal-cortical interface, potentially affecting meningeal and immune cell behaviour [166], prompting further investigation into how meningeal cells respond to AD pathology.

Recent data also point to disease-associated alterations in meningeal cell populations. Single-cell transcriptomic analyses demonstrate a marked reduction of perivascular fibroblasts in the AD cortex – opposite to the fibroblast expansion typically observed after acute CNS injury or inflammation – suggesting a distinct and attenuated cellular response in AD [167]. Additionally, several leptomeningeal cell types, including fibroblasts and immune cells, display altered gene expression signatures in AD patient tissue, and meningeal fibroblasts respond to Aβ in vitro in a comparable manner [86]. Such cellular changes may interact with local immune responses, which are also increasingly recognized as contributors to AD.

Perivascular and barrier-associated macrophages located in both the parenchyma and leptomeninges participate in Aβ clearance; their depletion leads to increased amyloid deposition [168]. Moreover, post-mortem studies suggest that extravascular T-cell accumulation in AD may reflect impaired CNS barrier function and chemotactic signals driven by amyloid and neuroinflammation. Such T-cell infiltration is proposed to amplify inflammatory cascades and increase neuronal vulnerability, potentially accelerating disease progression [169]. These findings underscore that meningeal immune responses, together with cellular meningeal and vascular alterations, may shape the inflammatory milieu in AD.

Taken together, emerging evidence suggests that meningeal dysfunction in AD involves multiple interconnected components – impaired lymphatic drainage, vascular and perivascular pathology, altered fibroblast states, and dysregulated immune cell activity. Although still incompletely understood, this multifaceted meningeal involvement may critically influence amyloid dynamics and neuroinflammatory processes. Future studies dissecting these pathways may help identify novel therapeutic entry points.

Overall, the meninges function as an active neuroimmune organ whose roles span barrier formation, immune surveillance and disease modulation. Recognizing this complexity will be crucial for understanding CNS pathology and for developing targeted interventions in the future.

## Data Availability

Not applicable.

## Abbreviations

AD: Alzheimer’s disease
Aβ: Amyloid beta
CNS: Central nervous system
CSF: Cerebrospinal fluid
EAE: Experimental autoimmune encephalomyelitis
mLVs: meningeal lymphatic vessels
MS: Multiple sclerosis
